# Supplementary material for: The social readjustment rating scale: Updated and modernised
Source: PLoS One. 2023 Dec 18;18(12):e0295943. doi: 10.1371/journal.pone.0295943 (PMC10727443; doi:10.1371/journal.pone.0295943)
Supplement: S1 Checklist — (DOCX) [file pone.0295943.s001.docx]

**The SRRS Updated and Modernised: STROBE Checklist**

|  | Item No. | Recommendation | Page  No. |
| --- | --- | --- | --- |
| **Title and abstract** | 1 | (*a*) Indicate the study’s design with a commonly used term in the title or the abstract | 1 |
|  |  | (*b*) Provide in the abstract an informative and balanced summary of what was done and what was found | 1 |
| Introduction | | | |
| Background/rationale | 2 | Explain the scientific background and rationale for the investigation being reported | 2-7 |
| Objectives | 3 | State specific objectives, including any prespecified hypotheses | 7-9 |
| Methods | | | |
| Study design | 4 | Present key elements of study design early in the paper | 9 |
| Setting | 5 | Describe the setting, locations, and relevant dates, including periods of recruitment, exposure, follow-up, and data collection | 10 |
| Participants | 6 | (*a*) *~~Cohort study~~*~~—Give the eligibility criteria, and the sources and methods of selection of participants. Describe methods of follow-up~~  *~~Case-control study~~*~~—Give the eligibility criteria, and the sources and methods of case ascertainment and control selection. Give the rationale for the choice of cases and controls~~  *Cross-sectional study*—Give the eligibility criteria, and the sources and methods of selection of participants | 10 |
|  |  | ~~(~~*~~b~~*~~)~~ *~~Cohort study~~*~~—For matched studies, give matching criteria and number of exposed and unexposed~~  *~~Case-control study~~*~~—For matched studies, give matching criteria and the number of controls per case~~ | n/a |
| Variables | 7 | Clearly define all outcomes, exposures, predictors, potential confounders, and effect modifiers. Give diagnostic criteria, if applicable | 11-13 |
| Data sources/ measurement | 8* | For each variable of interest, give sources of data and details of methods of assessment (measurement). Describe comparability of assessment methods if there is more than one group | 11-13 |
| Bias | 9 | Describe any efforts to address potential sources of bias | 12-14 |
| Study size | 10 | Explain how the study size was arrived at | 14-15 |

Continued on next page

|  | Item No. | Recommendation | Page  No. |
| --- | --- | --- | --- |
| Quantitative variables | 11 | Explain how quantitative variables were handled in the analyses. If applicable, describe which groupings were chosen and why | 12, 15, 45-46 |
| Statistical methods | 12 | (*a*) Describe all statistical methods, including those used to control for confounding | 14-15 |
|  |  | (*b*) Describe any methods used to examine subgroups and interactions | 14-15 |
|  |  | (*c*) Explain how missing data were addressed | 16 |
|  |  | (*d*) *~~Cohort study~~*~~—If applicable, explain how loss to follow-up was addressed~~  *~~Case-control study~~*~~—If applicable, explain how matching of cases and controls was addressed~~  *Cross-sectional study*—If applicable, describe analytical methods taking account of sampling strategy | 14 |
|  |  | (*e*) Describe any sensitivity analyses | 15 |
| Participants | 13* | (a) Report numbers of individuals at each stage of study—eg numbers potentially eligible, examined for eligibility, confirmed eligible, included in the study, completing follow-up, and analysed | 16 |
|  |  | (b) Give reasons for non-participation at each stage | 16 |
|  |  | (c) Consider use of a flow diagram | n/a |
| Descriptive data | 14* | (a) Give characteristics of study participants (eg demographic, clinical, social) and information on exposures and potential confounders | 16-17  Table 1 |
|  |  | (b) Indicate number of participants with missing data for each variable of interest | 16 |
|  |  | ~~(c)~~ *~~Cohort study~~*~~—Summarise follow-up time (eg, average and total amount)~~ | n/a |
| Outcome data | 15* | *~~Cohort study~~*~~—Report numbers of outcome events or summary measures over time~~ | n/a |
|  |  | *~~Case-control study—~~*~~Report numbers in each exposure category, or summary measures of exposure~~ | n/a |
|  |  | *Cross-sectional study—*Report numbers of outcome events or summary measures | 6 demographic outcomes (Table 1,5,6);  43 life event outcomes (Table 3,4,5,6,7);  43 life events grouped into 4 categories (Table 4)  2 loneliness outcomes |

|  | Item No. | Recommendation | Page  No. | |  |
| --- | --- | --- | --- | --- | --- |
| Main results | 16 | (*a*) Give unadjusted estimates and, if applicable, confounder-adjusted estimates and their precision (eg, 95% confidence interval). Make clear which confounders were adjusted for and why they were included | Table 1 (demographic predictors)  Table 3, Table 8 (Life events weights)  Table 7 (Personal experience of life events) | |  |
|  |  | (*b*) Report category boundaries when continuous variables were categorized | Table 4 (age: young, middle-aged, older adults)  Fig. 2 (life events outcomes grouped as: personal, work, family, financial) | |  |
|  |  | (*c*) If relevant, consider translating estimates of relative risk into absolute risk for a meaningful time period | n/a | |  |
| Other analyses | 17 | Report other analyses done—eg analyses of subgroups and interactions, and sensitivity analyses |  | 17-47 | |
| Discussion | | | | | |
| Key results | 18 | Summarise key results with reference to study objectives |  | 48 | |
| Limitations | 19 | Discuss limitations of the study, taking into account sources of potential bias or imprecision. Discuss both direction and magnitude of any potential bias |  | 53 | |
| Interpretation | 20 | Give a cautious overall interpretation of results considering objectives, limitations, multiplicity of analyses, results from similar studies, and other relevant evidence |  | 54-55 | |
| Generalisability | 21 | Discuss the generalisability (external validity) of the study results |  | 48, 54 | |
| Other information | |  | | | |
| Funding | 22 | Give the source of funding and the role of the funders for the present study and, if applicable, for the original study on which the present article is based |  | n/a | |
