# Supplementary material for: The social readjustment rating scale: Updated and modernised
Source: PLoS One. 2023 Dec 18;18(12):e0295943. doi: 10.1371/journal.pone.0295943 (PMC10727443; doi:10.1371/journal.pone.0295943)
Supplement: S1 Appendix — (PDF) [file pone.0295943.s002.pdf]

S1 Appendix 1

Social Readjustment Rating Questionnaire

The Social Readjustment Rating Scale

1. Social readjustment includes the amount and duration of change in one's accustomed pattern of life resulting from various life events. Social readjustment measures the intensity and length of time necessary for you to adapt, *regardless of the desirability of this event*.

2. *Use all of your own experience*, as well as what you have learned to be the case for others, in judging the amount of adjustment required. You need not have experienced something yourself to have an opinion about the amount of adjustment it would take.

3. Suppose that marriage takes 50 units of adjustment. Compare each of the events below to marriage and think to yourself, "Would this event require more or less adjustment than marriage?", "Would the readjustment take longer or shorter to accomplish?" If you decide the readjustment is more intense and protracted, enter a *proportionately larger* number than 50 for the event. If you decide the event represents less and shorter readjustment than marriage then indicate how much less by providing a *proportionately smaller* number next to the event. If it is equal to marriage, enter the number 50 next to the event.

Kindly use the above criteria to provide your chosen value for each of the events below relative to marriage, which is defined as 50.

Use any number between 0 - 100.

S1 Appendix 1

	Life Event	Rating
1	Troubles with the boss	________
2	Detention in jail or other institution	________
3	Death of a spouse or life partner	________
4	Major change in sleeping habits (a lot more or a lot less, or change in part of day when asleep)	________
5	Death of a close family member	________
6	Major change in eating habits (a lot more or a lot less food intake, or very different meal hours or surroundings)	________
7	Foreclosure/repossession on mortgage or loan	________
8	Revision of personal habits (dress, manners, associations, etc.)	________
9	Death of a close friend	________
10	Minor violations of the law (e.g. traffic ticket, disturbing the peace)	________
11	Outstanding personal achievement	________
12	Pregnancy (either yourself or being the father)	________
13	Major change in the health or behaviour of a family member	________
14	Sexual difficulties	________
15	In-law troubles	________
16	Major change in number of family get-togethers (e.g. a lot more or a lot less than usual)	________
17	Major change in financial state (e.g. a lot worse off or a lot better off than usual)	________
18	Gaining a new family member (e.g. through birth, adoption, grandparent moving in, etc.)	________
19	Change in residence	________
20	Son or daughter leaving home (e.g. marriage, attending college, etc.)	________
21	Marital separation	________
22	Major change in religious activities (e.g. a lot more or a lot less than usual)	________
23	Marital reconciliation	________
24	Losing your job (redundancy, dismissal, etc.)	________
25	Divorce	________
26	Changing to a different line of work	________
27	Major change in the number of arguments with spouse or life partner (e.g. either a lot more or a lot less than usual regarding child-rearing, personal habits, etc.)	________
28	Major change in responsibilities at work (e.g. promotion, demotion, lateral transfer)	________
29	Spouse or life partner begins or stops working	________
30	Major change in work hours or conditions	________
31	Major change in usual type and/or amount of recreation	________
32	Taking on a mortgage or loan for a major purchase (e.g. purchasing a home, business, etc.)	________
33	Taking on a loan for a lesser purchase (e.g. purchasing a car or furniture, paying for college fees, etc.)	________
34	Major personal injury or illness	________

S1 Appendix 1

	Life Event (cont'd.)	Rating
35	Major business readjustment (e.g. merger, reorganization, bankruptcy, etc.)	________
36	Major change in social activities (e.g. clubs, dancing, movies, visiting, etc.)	________
37	Major change in living conditions (e.g. building a new home, remodelling, deteriorating of home or neighbourhood)	________
38	Retirement from work	________
39	Vacation	________
40	Christmas	________
41	Changing to a new school	________
42	Beginning or ceasing formal schooling	________
43	Single person, living alone	________

REFERENCE:

Holmes, T. H., & David, E. M. (Eds.). (1989). *Life change, life events, and illness: selected papers*: Praeger Publishers.

SRRS item wording changes.

Original item wording	New item wording
Death of spouse	Death of a spouse or life partner
Minor violations of the law (e.g. traffic ticket, jay walking, disturbing the peace)	Minor violations of the law (e.g. traffic ticket, disturbing the peace)
Pregnancy	Pregnancy (either yourself or being the father)
Gaining a new family member (e.g. through birth, adoption, oldster moving in, etc.)	Gaining a new family member (e.g. through birth, adoption, grandparent moving in, etc.)
Marital separation from mate	Marital separation
Major change in church activities (e.g. a lot more or a lot less than usual)	Major change in religious activities (e.g. a lot more or a lot less than usual)
Marital reconciliation with mate	Marital reconciliation
Being fired from work	Losing your job (redundancy, dismissal, etc.)

S1 Appendix 1

Cont'd.

Original item wording	New item wording
Major change in the number of arguments with spouse (e.g. either a lot more or a lot less than usual regarding child-rearing, personal habits, etc.)	Major change in the number of arguments with spouse or life partner (e.g. either a lot more or a lot less than usual regarding child-rearing, personal habits, etc.)
Spouse begins or stops working outside the home	Spouse or life partner begins or stops working
Taking on a mortgage greater than $10,000 (e.g. purchasing a home, business, etc.)	Taking on a mortgage or loan for a major purchase (e.g. purchasing a home, business, etc.)
Taking on a mortgage or loan less than $10,000 (e.g. purchasing a car or furniture, paying for college fees, etc.)	Taking on a loan for a lesser purchase (e.g. purchasing a car or furniture, paying for college fees, etc.)
