# Supplementary material for: The social readjustment rating scale: Updated and modernised
Source: PLoS One. 2023 Dec 18;18(12):e0295943. doi: 10.1371/journal.pone.0295943 (PMC10727443; doi:10.1371/journal.pone.0295943)
Supplement: S2 Appendix — (PDF) [file pone.0295943.s003.pdf]

S2 Appendix 2

Social Readjustment Rating Questionnaire original instructions

The Social Readjustment Rating Scale instructions only (Holmes & Rahe, 1967, p. 213)

A. Social readjustment includes the amount and duration of change in one's accustomed pattern of life resulting from various life events. As defined, social readjustment measures the intensity and length of time necessary to accommodate to a life event, *regardless of the desirability of this event.*

B. You are asked to rate a series of life events as to their relative degrees of necessary readjustment. In scoring*, use all of your experience* in arriving at your answer. This means personal experience where it applies as well as what you have learned to be the case for others. Some persons accommodate to change more readily than others; some persons adjust with particular ease or difficulty to only certain events. Therefore, strive to give your opinion of the average degree of readjustment necessary for each event rather than the extreme.

C. The mechanics of rating are these : Event 1, Marriage, has been given an arbitrary value of 500. As you complete each of the remaining events think to yourself, "Is this event indicative of more -or less readjustment than marriage?" "Would the readjustment take longer or shorter to accomplish?" If you decide the readjustment is more intense and protracted, then choose a *proportionately larger* number and place it in the blank directly opposite the event in the column marked "VALUES." If you decide the event represents less and shorter readjustment than marriage then indicate how much less by placing a proportionately smaller number in the opposite blank. (If an event requires intense readjustment over a short time span, it may approximate in value an event requiring less intense readjustment over a long period of time.) If the event is equal in social readjustment to marriage, record the number 500 opposite the event.

REFERENCE:

Holmes, T. H., & Rahe, R. H. (1967). The social readjustment rating scale. *Journal of psychosomatic research*.
