# Supplementary material for: The social readjustment rating scale: Updated and modernised
Source: PLoS One. 2023 Dec 18;18(12):e0295943. doi: 10.1371/journal.pone.0295943 (PMC10727443; doi:10.1371/journal.pone.0295943)
Supplement: S3 Appendix — (PDF) [file pone.0295943.s004.pdf]

S3 Appendix 3

Updated SRRS and table showing changes to items

Social Readjustment Rating Scale 2022

Life Event	Yes	No
Marriage	O	O
Losing your job (e.g. redundancy, dismissal, etc.)	O	O
Change in religious activities (e.g. a lot more or a lot less than usual)	O	O
Revision of personal habits (e.g. dress, manners, associations)	O	O
Sexual difficulties	O	O
Trouble with in-laws	O	O
Major change in health or behaviour of family member	O	O
Taking on a mortgage or loan for a major purchase (e.g. home, business)	O	O
Taking on a loan for a lesser purchase (e.g. car, furniture)	O	O
Change in eating habits (e.g. a lot more or a lot less food intake, or very different meal hours or surroundings)	O	O
Pregnancy either yourself or being the father	O	O
Troubles with boss	O	O
Change in financial state (e.g. a lot worse off or a lot better off than usual)	O	O
Change to a different line of work	O	O
Marital reconciliation	O	O
Change in number of arguments with spouse/life partner (e.g. either a lot more or a lot less than usual regarding child-rearing, personal habits, etc.)	O	O
Change in living conditions (e.g. building new home, remodelling, deterioration of neighbourhood or home)	O	O

S3 Appendix 3

Outstanding personal achievement	O	O
Retirement	O	O
Business readjustment (e.g. merger, reorganisation, bankruptcy)	O	O
Spouse/life partner begins or stops work	O	O
Change in residence	O	O
Change in sleeping habits (e.g. a lot more or a lot less, or change in part of day when asleep)	O	O
Gain of new family member (e.g. through birth, adoption, grandparent moving in)	O	O
Change in work hours or conditions	O	O
Son or daughter leaving home (e.g. attend college, marriage)	O	O
Change in responsibilities at work (e.g. promotion, demotion, lateral transfer)	O	O
Change in social activities (e.g. clubs, dancing, movies, visiting)	O	O
Divorce	O	O
Personal injury or illness	O	O
Death of close family member	O	O
Change in recreation type/amount	O	O
Death of spouse/life partner	O	O
Change in number of family get-togethers (e.g. a lot more or a lot less than usual)	O	O
Detention in jail or other institution	O	O
Marital separation	O	O
Vacation	O	O

S3 Appendix 3

Foreclosure/repossession on mortgage or loan	O	O
Death of close friend	O	O
Changing to a new school	O	O
Begin or end formal schooling	O	O
Christmas	O	O
Minor violations of the law (e.g. traffic/parking tickets)	O	O
Single person, living alone	O	O

S3 Appendix 3

<u>Changed items</u>:

Original item rank	Original Item wording	New item wording
1	Death of spouse	Death of spouse/life partner
4	Jail term	Detention in jail or other institution
8	Fired at work	Losing your job (e.g. redundancy, dismissal, etc.)
11	Change in health of family member	Major change in health or behaviour of family member
12	Pregnancy	Pregnancy either yourself or being the father
13	Sex difficulties	Sexual difficulties
14	Gain of new family member	Gain of new family member (e.g. through birth, adoption, grandparent moving in)
15	Business readjustment	Business readjustment (e.g. merger, reorganisation, bankruptcy)
16	Change in financial state	Change in financial state (e.g. a lot worse off or a lot better off than usual)
19	Change in number of arguments with spouse	Change in number of arguments with spouse/life partner (e.g. either a lot more or a lot less than usual regarding child-rearing, personal habits, etc.)
20	Mortgage over $10,000	Taking on a mortgage or loan for a major purchase (e.g. home, business)
21	Foreclosure of mortgage or loan	Foreclosure/repossession on mortgage or loan
22	Change in responsibilities at work	Change in responsibilities at work (e.g. promotion, demotion, lateral transfer)
23	Son or daughter leaving home	Son or daughter leaving home (e.g. attend college, marriage)
26	Wife begin or stop work	Spouse/life partner begins or stops work
27	Begin or end schooling	Begin or end formal schooling
28	Change in living conditions	Change in living conditions (e.g. building new home, remodelling, deterioration of neighbourhood or home)
29	Revision of personal habits	Revision of personal habits (e.g. dress, manners, associations)
33	Change in schools	Changing to a new school
34	Change in recreation	Change in recreation type/amount
35	Change in church activities	Change in religious activities (e.g. a lot more or a lot less than usual)
36	Change in social activities	Change in social activities (e.g. clubs, dancing, movies, visiting)
37	Mortgage of loan less than $10,000	Taking on a loan for a lesser purchase (e.g. car, furniture)
38	Change in sleeping habits	Change in sleeping habits (e.g. a lot more or a lot less, or change in part of day when asleep)
39	Change in number of family get-togethers	Change in number of family get-togethers (e.g. a lot more or a lot less than usual)
40	Change in eating habits	Change in eating habits (e.g. a lot more or a lot less food intake, or very different meal hours or surroundings)
43	Minor violations of the law	Minor violations of the law (e.g. traffic/parking tickets)
