# Supplementary material for: The social readjustment rating scale: Updated and modernised
Source: PLoS One. 2023 Dec 18;18(12):e0295943. doi: 10.1371/journal.pone.0295943 (PMC10727443; doi:10.1371/journal.pone.0295943)
Supplement: S4 Appendix — (PDF) [file pone.0295943.s005.pdf]

## S4 Appendix 4

### Personal experience of SRRS life events

At the start of this survey you were asked to rate a range of life events by comparing them to marriage. To what extent was your chosen rating based on your own personal experience?

Please slide the scale to indicate as best you can how much your rating was based on your own experience from '***not at all based on my own experience***' **(0)** to '***completely based on my own experience***' **(100)**.

**S4 Appendix 4**

| | Not at all based on my own experience | About half | Completely based on my own experience |
|---|---|---|---|
| | 0 10 20 30 | 40 50 60 | 70 80 90 100 |
| Troubles with the boss | | | |
| Detention in jail or other institution | | | |
| Death of a spouse or life partner | | | |
| Major change in sleeping habits (a lot more or a lot less, or change in part of day when asleep) | | | |
| Death of a close family member | | | |
| Major change in eating habits (a lot more or a lot less food intake, or very different meal hours or surroundings) | | | |
| Foreclosure/repossession on mortgage or loan | | | |
| Revision of personal habits (dress, manners, associations, etc.) | | | |
| Death of of a close friend | | | |
| Minor violations of the law (e.g. traffic ticket, disturbing the peace) | | | |
| Outstanding personal achievement | | | |
| Pregnancy (either yourself or being the father) | | | |
| Major change in the health or behaviour of a family member | | | |
| Sexual difficulties | | | |
| In-law troubles | | | |
| Major change in number of family get-togethers (e.g. a lot more or a lot less than usual) | | | |
| Major change in financial state (e.g. a lot worse off or a lot better off than usual) | | | |

## S4 Appendix 4

| | |
|---|---|
| Gaining a new family member (e.g. through birth, adoption, grandparent moving in, etc.) | |
| Change in residence | |
| Son or daughter leaving home (e.g. marriage, attending college, etc.) | |
| Marital separation | |
| Major change in religious activities (e.g. a lot more or a lot less than usual) | |
| Marital reconciliation | |
| Losing your job (made redundant, dismissed, etc.) | |
| Divorce | |
| Changing to a different line of work | |
| Major change in the number of arguments with spouse/life partner (e.g. either a lot more or a lot less than usual regarding child-rearing, personal habits, etc.) | |
| Major change in responsibilities at work (e.g. promotion, demotion, lateral transfer) | |
| Spouse/life partner begins or stops working | |
| Major change in work hours or conditions | |
| Major change in usual type and/or amount of recreation | |
| Taking on a mortgage or loan for a major purchase (e.g. purchasing a home, business, etc.) | |
| Taking on a loan for a lesser purchase (e.g. car, furniture) | |
| Major personal injury or illness | |
| Major business readjustment (e.g. merger, reorganization, bankruptcy,etc.) | |
| Major change in social activities (e.g. clubs, dancing, movies, visiting, etc.) | |
| Major change in living conditions (e.g. building a new home, remodelling, deteriorating of home or neighbourhood) | |
| Retirement from work | |

**S4 Appendix 4**

| | |
|---|---|
| Vacation | 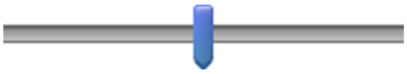 |
| Christmas | 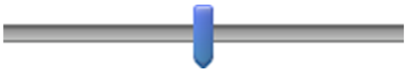 |
| Changing to a new school | 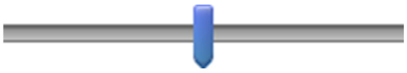 |
| Beginning or ceasing formal schooling | 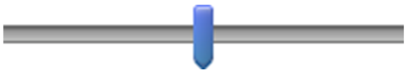 |
| Single person, living alone | 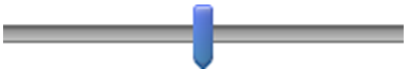 |
