# Supplementary material for: The social readjustment rating scale: Updated and modernised
Source: PLoS One. 2023 Dec 18;18(12):e0295943. doi: 10.1371/journal.pone.0295943 (PMC10727443; doi:10.1371/journal.pone.0295943)
Supplement: S5 Appendix — (PDF) [file pone.0295943.s006.pdf]

**S5 Appendix 5**

## Loneliness questionnaire

ONS Website contents *verbatim (Robards, 2022)*:

"Specifically, we recommend four questions to capture different aspects of loneliness. The first three questions are from the University of California, Los Angeles (UCLA) (Russell, 1996) three-item loneliness scale (Hughes, Waite, Hawkley, & Cacioppo, 2004). The wording of the UCLA questions and response options are taken from the English Longitudinal Study of Ageing (Lee et al., 2021). The last is a direct question about how often the respondent feels lonely, currently used on the Community Life Survey. For those aged 16 years and over, the loneliness measures should be as in Table 1."

**Table 1: Recommended measures of loneliness for adults**

| Measures | Items | Response categories |
|---|---|---|
| **The three-item UCLA Loneliness scale** | 1. How often do you feel that you lack companionship? | Hardly ever or never, Some of the time, Often |
| | 2. How often do you feel left out? | Hardly ever or never, Some of the time, Often |
| | 3. How often do you feel isolated from others? | Hardly ever or never, Some of the time, Often |
| **The direct measure of loneliness** | How often do you feel lonely? | Often/always, Some of the time, Occasionally, Hardly ever, Never |

Source: Office for National Statistics (Robards, 2022)
[https://www.ons.gov.uk/peoplepopulationandcommunity/wellbeing/methodologies/measuringlonelinessguidanceforuseofthenationalindicatorsonsurveys]

R-UCLA Scoring:

Items 1 – 3: Hardly ever or never = 1, Some of the time = 2, Often = 3

A higher score indicates a greater degree of loneliness.

**S5 Appendix 5**

REFERENCES:

Hughes, M. E., Waite, L. J., Hawkley, L. C., & Cacioppo, J. T. (2004). A Short Scale for Measuring Loneliness in Large Surveys: Results From Two Population-Based Studies. *Res Aging, 26*(6), 655-672. doi:10.1177/0164027504268574

Lee, S. L., Pearce, E., Ajnakina, O., Johnson, S., Lewis, G., Mann, F., . . . Lewis, G. (2021). The association between loneliness and depressive symptoms among adults aged 50 years and older: a 12-year population-based cohort study. *Lancet Psychiatry, 8*(1), 48-57. doi:10.1016/s2215-0366(20)30383-7

Robards, J. (2022). *Principal projection for the UK - population by five-year age groups and sex.* ONS website: Office for National Statistics Retrieved from https://www.ons.gov.uk/peoplepopulationandcommunity/wellbeing/methodologies/measuringlonelinessguidanceforuseofthenationalindicatorsonsurveys

Russell, D. W. (1996). UCLA Loneliness Scale (Version 3): reliability, validity, and factor structure. *J Pers Assess, 66*(1), 20-40. doi:10.1207/s15327752jpa6601_2
