# Supplementary material for: The social readjustment rating scale: Updated and modernised
Source: PLoS One. 2023 Dec 18;18(12):e0295943. doi: 10.1371/journal.pone.0295943 (PMC10727443; doi:10.1371/journal.pone.0295943)
Supplement: S6 Appendix — (PDF) [file pone.0295943.s007.pdf]

S6 Appendix 6

Table. Robustness Checks for all Mann-Whitney U Bayesian Analyses

		Cauchy value			
		userPrior (medium)	wide prior	ultrawide prior	
**Variable of interest**	**Comparison**	**0.707**	**1.000**	**1.414**	**N**
	overall SRRS weights original sample vs. new sample	6.22	5.08	3.64	86
	Wording change weight difference (new - original)	0.35	0.29	0.21	43
	Normative sample vs. 'old-older' adults	0.24	0.17	0.12	86
age group	YA vs. MA	0.23	0.16	0.12	86
	YA vs. OA	0.22	0.16	0.12	86
	MA vs. OA	0.23	0.16	0.12	86
sex	Males vs. Females	1.42	1.22	0.90	86
age group (YA vs. MA)	family	0.21	0.11	0.08	407
	financial	0.26	0.14	0.10	407
	personal	0.13	0.09	0.06	407
	work	0.13	0.09	0.06	407
age group (YA vs. OA)	family	0.16	0.11	0.08	249
	financial	0.14	0.10	0.07	249
	personal	0.15	0.11	0.08	249
	work	0.14	0.11	0.08	249
age group (MA vs. OA)	family	0.11	0.08	0.06	424
	financial	0.19	0.16	0.11	424
	personal	0.12	0.08	0.06	424
	work	0.11	0.08	0.06	424
sex (m vs. f)	family	73382.37	57806.85	3206.45	538
	financial	27.22	30.46	18.36	538
	personal	4039.19	3507.24	3606.85	538
	work	367.71	2189.98	3213.54	538
ethnicity (white vs. non-white)	family	0.13	0.08	0.06	540
	financial	0.20	0.08	0.06	540
	personal	0.13	0.08	0.06	540
	work	0.12	0.07	0.05	540
religion (no religion vs. religion)	family	0.24	0.16	0.12	540
	financial	0.27	0.19	0.15	540
	personal	0.59	0.51	0.38	540
	work	0.28	0.22	0.16	540
relationship status (married vs. unmarried)	family	1.74	1.61	1.14	540
	financial	0.11	0.08	0.06	540
	personal	0.16	0.15	0.11	540
	work	0.13	0.12	0.09	540
employment status (employed vs. unemployed)	family	0.20	0.12	0.09	540
	financial	0.11	0.08	0.06	540
	personal	0.10	0.07	0.05	540
	work	0.15	0.12	0.09	540
single person, living alone	age group (YA vs. MA)	0.17	0.11	0.08	407
	age group (YA vs. OA)	0.43	0.34	0.26	249
	age group (MA vs. OA)	0.16	0.10	0.13	424
	sex (males vs. females)	0.52	0.34	0.25	538
	ethnicity (white vs. non-white)	0.14	0.10	0.07	540
	religion (religion vs. no religion)	1.68	0.99	0.71	540
	relationship status (married vs. not married)	1.89	1.39	1.01	540
	employment status (employed vs. unemployed)	0.12	0.09	0.06	540

This table provides sensitivity anayses for all between-subjects comparisons and the respective sample size used in each case. Sensitivity analyses were performed by adjusting the the Cauchy distribution which changes the likelihood of capturing evidence of an effect.
