# Supplementary material for: The social readjustment rating scale: Updated and modernised
Source: PLoS One. 2023 Dec 18;18(12):e0295943. doi: 10.1371/journal.pone.0295943 (PMC10727443; doi:10.1371/journal.pone.0295943)
Supplement: S7 Appendix — (PDF) [file pone.0295943.s008.pdf]

S7 Appendix 7

Social Readjustment Rating Scale by category (Rahe, 1975, p. 251)

Family

1. Death of a spouse
2. Divorce
3. Marital separation
4. Death of close family member
5. Marriage
6. Marital reconciliation
7. Major change in health of family
8. Pregnancy
9. Major change in arguments w/wife
10. Son/daughter leaving home
11. In-law troubles
12. Wife starting or ending work
13. Major change in family get-togethers
14. Addition of new family member

Personal

15. Detention in jail
16. Major personal injury or illness
17. Sexual difficulties
18. Death of a close friend
19. Outstanding personal achievement
20. Start or end of formal schooling
21. Major change in living conditions
22. Major revision of personal habits
23. Changing to a new school
24. Change in residence
25. Major change in recreation
26. Major change in church activities
27. Major change in sleeping habits
28. Major change in eating habits
29. Vacation
30. Christmas
31. Minor violations of the law

Work

32. Being fired from work
33. Retirement from work
34. Major business adjustment
35. Changing to a different line of work
36. Major change in work responsibilities
37. Trouble with the boss
38. Major change in working conditions

Financial

39. Major change in financial state
40. Mortgage or loan over 10,000
41. Mortgage foreclosure
42. Mortgage or loan less than 10,000

S7 Appendix 7

REFERENCE:

Rahe, R. H. (1975). Life changes and near-future illness reports. In L. Levi & U. S. v. U. S. Euler (Eds.), *Emotions - Their Parameters and Measurement.* (pp. 511-529). New York: Raven Press.
