# Supplementary material for: The social readjustment rating scale: Updated and modernised
Source: PLoS One. 2023 Dec 18;18(12):e0295943. doi: 10.1371/journal.pone.0295943 (PMC10727443; doi:10.1371/journal.pone.0295943)
Supplement: S8 Appendix — (PDF) [file pone.0295943.s009.pdf]

S8 Appendix 8

Table. Descriptive statistics for SRRS events by unabridged sub-group demographics, categorised as family, financial, personal or work items.

		Mean SRRS weights			
		family items	financial items	personal items items	work items
Age	sub-groups	Median (IQR)	Median (IQR)	Median (IQR)	Median (IQR)
	< 30 years	53.6 (44.8 - 61)	50 (33.8 - 55.8)	35.5 (28.6 - 45.3)	42.1 (31.6 - 54.3)
	30 to 60 years	54.6 (45.7 - 64.3)	45 (35 - 52.5)	34.7 (28.9 - 43.3)	43.6 (32.1 - 52.9)
	> 60 years	53.9 (46.1 - 61.1)	46.5 (35.6 - 58.8)	33.4 (26.4 - 46.6)	42.9 (33.2 - 54.7)
Sex					
	female	57.1 (48.9 - 64.9)	47.5 (37.5 - 56.2)	36.9 (30.6 - 46.5)	45.7 (35.7 - 55.7)
	male	50.4 (42.4 - 58.8)	42.5 (30 - 55)	32.3 (24.7 - 40.5)	40 (29.3 - 50)
ethnicity					
	white	54.3 (46.4 - 61.8)	46.3 (35 - 55)	34.2 (27.9 - 43.7)	42.9 (32.9 - 52.9)
	mixed race	50.4 (39.8 - 59.6)	41.3 (33.8 - 60)	38.2 (30.8 - 44.5)	43.9 (30.7 - 50)
	asian (southern/southeastern asia)	50.7 (37 - 60.6)	42.5 (23 - 58.4)	30.6 (19 - 44.8)	43.6 (23.5 - 54.3)
	chinese (east asian)	58.6 (55.4 - 67.1)	50 (35 - 52.5)	35.3 (32.1 - 44.5)	48.6 (35.7 - 57.1)
	black (any region)	60.4 (46.8 - 67.9)	52.5 (42.5 - 65)	44.7 (32.6 - 49.8)	51.4 (40 - 60.1)
religion					
	no religion	53.6 (45.6 - 60.7)	45 (35 - 54.1)	33.9 (28 - 41.1)	42.1 (32.1 - 52.1)
	Christian	63 (43.7 - 73.3)	52.5 (26.9 - 77.2)	43.7 (27.1 - 48.2)	47.1 (32 - 53.2)
	Buddhist	55.7 (46.4 - 64.4)	47.5 (35 - 57.5)	36.1 (28.3 - 47.1)	44.3 (34.3 - 55.3)
	Hindu	49.6 (31.8 - 59.1)	58.8 (27.5 - 83.1)	32.8 (19.6 - 47.2)	45.7 (25.4 - 60.1)
	Jewish	48.7 (36.2 - 56.9)	38.1 (30.6 - 42.5)	28.1 (20.9 - 36.4)	37.5 (28.1 - 45.7)
	Muslim	50.4 (31.5 - 62.2)	42.5 (20.3 - 55)	33.7 (16.6 - 45.5)	32.9 (18.8 - 45.4)
	Sikh	51.8 (44.9 - 72)	58.8 (36.9 - 66.3)	28.7 (25.1 - 62.2)	53.6 (32.9 - 75.7)
	any other religion	55 (46.4 - .)	52.5 (42.5 - .)	31.6 (26.9 - .)	48.6 (35.7 - .)
relationship status					
	divorced	55 (45.9 - 58.9)	47.5 (35.6 - 56.3)	33.9 (31 - 41.7)	47.9 (34.3 - 52.5)
	in a relationship	50.5 (43.1 - 57.1)	41.3 (32.8 - 51.8)	34.3 (25.9 - 40.7)	38.2 (28.4 - 47)
	married/LTR	55 (46.8 - 64.5)	46.5 (35 - 55)	34.7 (28.4 - 45.3)	42.9 (32.9 - 54.3)
	separated	40.8 (16.9 - 54.4)	65 (10.6 - 69.8)	28 (17.4 - 40.3)	31.4 (14.3 - 51.4)
	single	54.3 (44.3 - 61.8)	46.3 (34.4 - 55.6)	35.8 (27.1 - 44.7)	44.3 (32.9 - 53.9)
	widowed/LP died	50.4 (45 - 58.8)	46.3 (35.3 - 59.7)	29.9 (28.2 - 48.8)	44.6 (29.5 - 52.5)
employment status					
	currently unemployed, looking for work	53.9 (43.9 - 63.5)	49.4 (41.3 - 56.9)	31.4 (26.2 - 38.2)	42.1 (31.4 - 54.1)
	full-time or part-time employed	54.3 (46.1 - 63)	46 (35 - 55)	34.7 (28.2 - 44.5)	42.9 (32.9 - 55)
	long-term sick or disabled	61.1 (57.9 - 67.4)	52.5 (47.5 - 65)	41.6 (36.9 - 49.5)	49.3 (45.7 - 61.4)
	looking after home or family	58.3 (50.8 - 64.4)	47.5 (37.8 - 52.5)	36.1 (32.1 - 42.9)	43.6 (32.3 - 50.5)
	other*	47.1 (40 - 55.7)	35 (28.1 - 50.6)	34.5 (22.1 - 38.7)	32.9 (27.9 - 54.6)
	retired	51.6 (44.1 - 59.3)	45 (33.8 - 58.1)	32.2 (25.3 - 46.1)	41.4 (29.6 - 52.4)
	student (p/t or f/t) and currently unemployed	51.1 (44.7 - 60.4)	48.8 (33.8 - 57.5)	37.4 (31.3 - 47.6)	44.3 (35 - 54.3)

*other combines 'have never worked' (n=2) and the entry where 1 person gave no specific details.
