# Supplementary material for: The social readjustment rating scale: Updated and modernised
Source: PLoS One. 2023 Dec 18;18(12):e0295943. doi: 10.1371/journal.pone.0295943 (PMC10727443; doi:10.1371/journal.pone.0295943)
Supplement: S9 Appendix — (PDF) [file pone.0295943.s010.pdf]

S9 Appendix 9

Table. Bayesian Kendall's tau correlation between event ratings and degree to which these were based on personal experience.

Rating vs. Personal experience of event	Kendall tau B	Lower 95% CI[a]	Upper 95% CI[a]	BF_{10}
Death of a close family member	0.146	0.09	0.20	19900.33
Detention in jail or other institution	-0.137	-0.19	-0.08	4564.74
Major change in the health or behaviour of a family member	0.135	0.08	0.19	3109.20
Major change in social activities	0.128	0.07	0.18	951.76
Major change in religious activities	0.125	0.07	0.18	672.81
Foreclosure/repossession on mortgage or loan	-0.108	-0.16	-0.05	62.30
Major change in sleeping habits	0.091	0.03	0.15	7.84
Major change in eating habits	0.089	0.03	0.15	6.53
Retirement from work	-0.088	-0.15	-0.03	6.00
Major change in usual type and/or amount of recreation	0.088	0.03	0.14	5.71
Major business readjustment	-0.084	-0.14	-0.03	4.00
Gaining a new family member	0.084	0.03	0.14	3.73
Revision of personal habits	0.083	0.03	0.14	3.33
Major change in number of family get-togethers	0.082	0.03	0.14	3.19
Sexual difficulties	0.081	0.03	0.14	3.00
Changing to a new school	0.077	0.02	0.13	1.98
Beginning or ceasing formal schooling	0.077	0.02	0.13	1.90
Change in residence	0.065	0.01	0.12	0.68
In-law troubles	0.063	0.01	0.12	0.61
Major change in the number of arguments with spouse-life partner	0.054	0.00	0.11	0.32
Troubles with the boss	0.053	0.00	0.11	0.31
Minor violations of the law	-0.05	-0.11	0.01	0.25
Vacation	-0.048	-0.11	0.01	0.22
Son or daughter leaving home	-0.047	-0.10	0.01	0.21
Spouse/life partner begins or stops working	-0.045	-0.10	0.01	0.19
Major change in financial state	0.042	-0.02	0.10	0.16
Outstanding personal achievement	0.037	-0.02	0.09	0.13
Marital reconciliation	-0.037	-0.09	0.02	0.13
Losing your job	-0.036	-0.09	0.02	0.12
Death of a close friend	-0.034	-0.09	0.02	0.11
Pregnancy	-0.03	-0.09	0.03	0.10
Changing to a different line of work	-0.029	-0.09	0.03	0.09
Christmas	-0.029	-0.09	0.03	0.09
Marital separation	0.025	-0.03	0.08	0.08
Major change in living conditions	0.023	-0.03	0.08	0.08
Major personal injury or illness	0.021	-0.04	0.08	0.08
Major change in responsibilities at work	0.019	-0.04	0.08	0.07
Death of a spouse or life partner	0.014	-0.04	0.07	0.06
Divorce	0.014	-0.04	0.07	0.06
Taking on a loan for a lesser purchase	-0.012	-0.07	0.05	0.06
Single person, living alone	-0.008	-0.07	0.05	0.06
Taking on a mortgage or loan for a major purchase	-0.003	-0.06	0.05	0.06
Major change in work hours or conditions	-0.004	-0.06	0.05	0.06

Bayes Factors (BFs) above the upper dotted line support H1 (that ratings correlate with personal experience).

BFs below the lower dotted line support H0 (that ratings do not correlate with personal experience).

BFs between the dotted lines support neither H1 nor H0 (i.e. evidence is inconclusive).

[a] CI = Credible Interval

N = 536

S9_Appendix9_personalExpBFcorrelations.xlsx
